# A Practical Guide to Incorporating Novel Barrett’s Screening/Surveillance Tools into Clinical Practice

**DOI:** 10.1007/s11894-026-01041-6

**Published:** 2026-04-06

**Authors:** Brian E. White, Alexandra L. Strauss Starling

**Affiliations:** 1 Department of Medicine, Hospital of the University of Pennsylvania, University of Pennsylvania Perelman School of Medicine, 3400 Civic Center Boulevard, Philadelphia, PA 19104-4311 USA; 2 Division of Gastroenterology and Hepatology, Department of Medicine, Hospital of the University of Pennsylvania, University of Pennsylvania, Perelman School of Medicine, Philadelphia, PA USA

**Keywords:** Barrett’s Esophagus Screening, Risk Stratification, Cell Collection Device, Serum Biomarkers, Liquid Biopsy, Methylated DNA Markers

## Abstract

**Purpose of Review:**

Barrett’s esophagus (BE) is the only known precursor for esophageal adenocarcinoma. Patients diagnosed in endoscopy-based screening and surveillance programs have improved outcomes, but there are many limitations with current screening and surveillance paradigms. This review examines novel technologies aimed at improving current BE screening and surveillance strategies.

**Recent Findings:**

BE risk stratification algorithms have decent discriminatory performance but are cumbersome to implement. Non-endoscopic screening tools, including swallowable cell collection devices, have shown good sensitivity and cost-effectiveness. Early studies of serum biomarkers, such as microRNA assays, have yielded promising results but warrant further validation. WATS-3D appears to increase dysplasia yield among patients undergoing surveillance. Tissue-based biomarkers may assist in risk stratification.

**Summary:**

Novel, non-endoscopic technologies have the potential to enrich the BE screening population and improve outcomes, although most require further validation. Collaboration with primary care providers is critical to maximize the impact of such interventions.

## Introduction

 The incidence of esophageal adenocarcinoma (EAC) has increased over the last several decades and continues to have a poor prognosis, with an overall 5 year survival rate < 20% for advanced stage disease [1, 2]. Barrett’s esophagus (BE) is the only known EAC precursor and the target for screening and surveillance programs [3]. Patients with prior BE have improved outcomes due to the detection of earlier stage disease, even after adjustment for lead-time bias [4–6]. Endoscopic eradication therapies (EET) are effective for patients with dysplasia and early EAC, conferring similar survival and lower morbidity than esophagectomy [7–10].

Gastroenterology professional societies recommend screening with upper endoscopy (EGD) in patients at risk for BE/EAC, as well as endoscopic surveillance [11–16]. Patients are considered at risk for BE/EAC in the presence of various clinical and demographic risk factors. EET is recommended for patients with high-grade dysplasia (HGD) or early EAC; EET is suggested for patients with low-grade dysplasia (LGD), although surveillance is a reasonable alternative [12, 17].

**Fig. 1:**
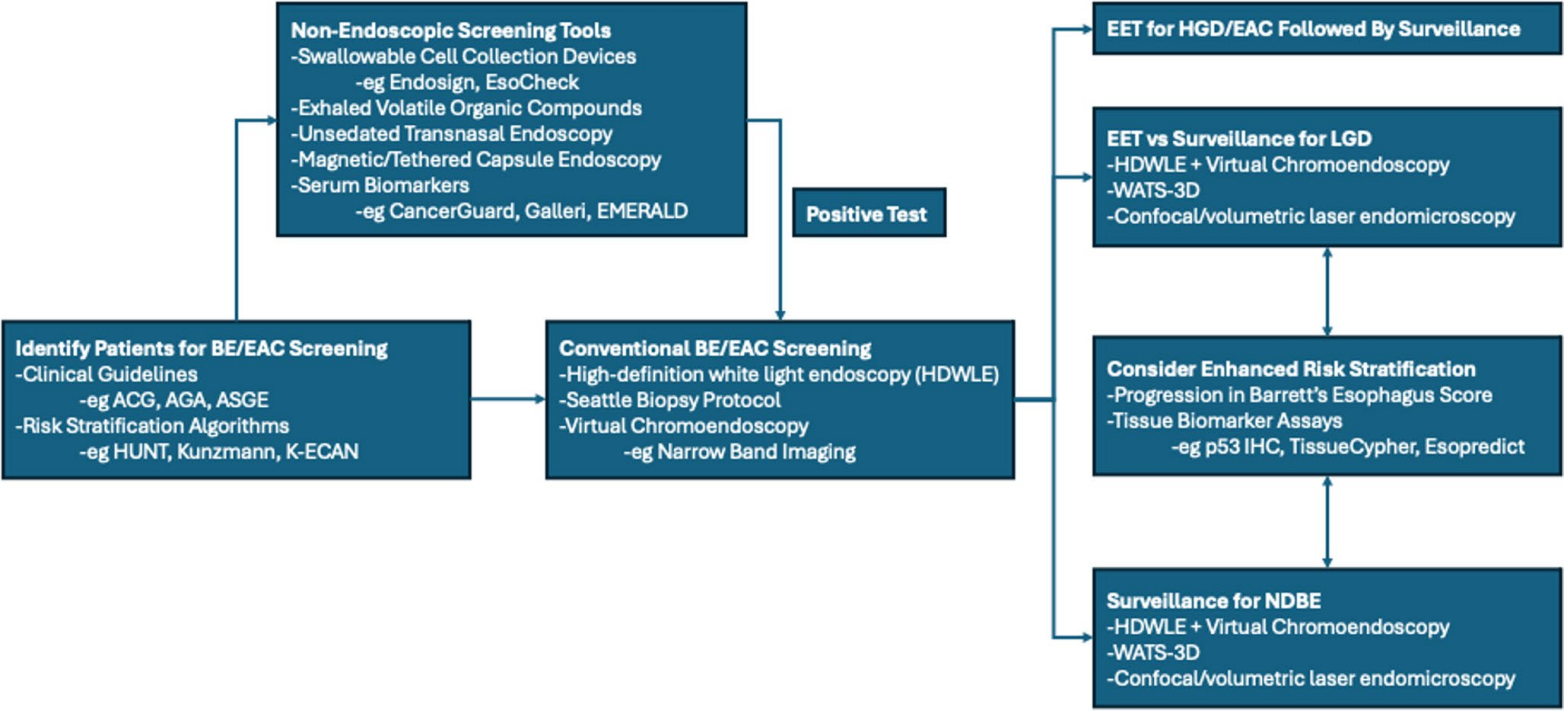
Elements of process for BE/EAC screening and surveillance, incorporating opportunities for novel tools to assist in initial risk assessment, non-endoscopic screening, detection of dysplasia, and risk stratification for patients under surveillance. BE, Barrett’s esophagus; EAC, esophageal adenocarcinoma; ACG, American College of Gastroenterology; AGA, American Gastroenterological Association; ASGE, American Society for Gastrointestinal Endoscopy; HGD, high-grade dysplasia; LGD, low-grade dysplasia; WATS-3D, Wide Area Transepithelial Sampling with 3-dimensional Computer-Assisted Analysis; IHC, immunohistochemistry; HDWLE, high-definition white light endoscopy; EET, endoscopic eradication therapy; HUNT, Nord-Trøndelag Health Study; K-ECAN, Kettles Esophageal and Cardia Adenocarcinoma prediction tool

About 88% of patients lack identification of BE prior to EAC diagnosis [18]. Multiple factors have been implicated in these missed opportunities. The emphasis on GERD may compromise the sensitivity of screening guidelines, as up to 50% of patients with EAC lack GERD symptoms [19]. Poor operationalization of guidelines, limited uptake by primary care providers, and the reliance on sedated EGD, an expensive and invasive procedure, have also been cited [20, 21]. Finally, a paucity of reliable methods to predict progression among patients with BE contributes to both over- and under-surveillance. This article will review novel technologies aimed at addressing the limitations of current BE screening and surveillance strategies, summarized in Fig. 1.

## Enriching the Barrett’s Esophagus Screening Population

The limitations raised by BE screening guidelines introduce the need for other methods to enrich the screening population. Current guidelines from the American College of Gastroenterology (ACG), American Society for Gastrointestinal Endoscopy (ASGE), British Society of Gastroenterology (BSG), and European Society of Gastrointestinal Endoscopy (ESGE) suggest BE screening in patients with chronic GERD and risk factors, including male sex, age > 50, white race, obesity, tobacco smoking, and a family history of BE/EAC [11, 12, 14, 16]. The requirement of GERD as a screening criterion limits sensitivity for BE/EAC, as 30–50% of patients with BE/EAC lack chronic GERD symptoms [12, 22]. Nguyen et al. determined that the sensitivities of the above guidelines for BE were poor, at 38.6%-43.2% [22]. The 2011 AGA guidelines, which did not require GERD but suggested BE screening for any 2 risk factors, were 100% sensitive but only 0.2% specific.

## Risk Prediction Tools for Targeted BE/EAC Screening

To improve screening performance, researchers have developed risk algorithms incorporating clinical, demographic, and anthropometric variables to predict BE and/or EAC. Importantly, these models do not assign equal weights to risk factors that vary in their strength of association with BE and EAC, and most do not require GERD [23, 24]. These tools, including the M-BERET, Kunzmann, HUNT, MARK-BE, Houston-BEST, and K-ECAN, have demonstrated variable discrimination for BE and related neoplasia, with areas under the receiver operating curve (AUROC) 0.66–0.87 (Table 1) [25–34]. Currently, risk algorithms remain limited by barriers such as cumbersome manual input and are not recommended by gastroenterology societies, although AGA guidelines note that they can be considered in the evaluation of patients for screening [13]. Further studies to evaluate aspects such as screening thresholds and EHR integration are warranted [35]. For now, publicly available risk algorithms that use variables available in the EHR, such as the Kunzmann and HUNT tools, may enhance risk assessment and an individualized approach to BE screening.

**Table 1:** Barrett’s esophagus and esophageal adenocarcinoma risk prediction tools

Risk Tool	Predictors	Outcome	Reference(s)	AUROC
M-BERET	GERD, age, waist-to-hip ratio, smoking	BE	Rubenstein et al [25]	0.72
		BE	Thrift et al [26]	0.70-0.72
		BE/early neoplasia*	Rubenstein et al [27]	0.70/0.77
		EAC/EGJAC	Rubenstein et al [28]	0.68/0.66
Kunzmann	Age, sex, smoking, BMI, esophageal condition(s)	EAC	Kunzmann et al [29]	0.80
		BE/early neoplasia*	Rubenstein et al [27]	0.67/0.76
		EAC/EGJAC	Rubenstein et al [28]	0.73/0.68
HUNT	Age, sex, GERD, BMI, smoking	10yr EAC/15yr EAC	Xie et al [30]	0.71/0.84
		BE/early neoplasia*	Rubenstein et al [27]	0.66/0.80
		EAC/EGJAC	Rubenstein et al [28]	0.66/0.65
MARK-BE	Age, sex, waist circumference, acid medications, cigarettes, heartburn, acid taste, stomach pain	BE	Rosenfeld et al [31]	0.81-0.87
Houston-BEST	Sex, age, race/ethnicity, smoking, BMI, GERD, first degree relative with EAC	BE	Wenker et al [32]	0.68-0.70
Iyer	>400 variables	BE/EAC	Iyer et al [33]	0.84/0.84
K-ECAN	211 variables	EAC	Rubenstein et al [34]	0.77
		EAC I/EAC II	Rubenstein et al [34]	0.81/0.79

BMI, body mass index; BE, Barrett’s esophagus; EAC, esophageal adenocarcinoma; EAC I, stage I EAC; EAC II, stage II EAC; EGJAC, esophagogastric junction adenocarcinoma; AUROC, area under the receiver operating characteristic curve; M-BERET, Michigan Barrett’s Esophagus pREdiction Tool; HUNT, Nord-Trøndelag Health Study; MARK-BE, Machine Learning Risk Prediction in Barrett’s Esophagus; Houston-BEST, Houston Barrett’s Electronic Screening Tool; K-ECAN, Kettles Esophageal and Cardia Adenocarcinoma prediction tool

* Early neoplasia was defined as T1a EAC, HGD, or LGD confirmed on expert pathology review

## Novel Barrett’s Esophagus Screening Tools

### Swallowable Cell Collection Devices

EGD with forceps biopsy (FB) remains the gold standard for diagnosis of BE and associated neoplasia; however, the procedure’s expense, need for sedation, risks, time investment, and limited endoscopist and institutional capacities pose barriers to use as a screening tool. The potential expansion of the screening population to include patients without GERD further underscores the need for alternative, cost-effective methods. Novel BE screening tools are summarized in Table 2. Swallowable cell collection devices represent the most well-developed category of non-endoscopic screening technology. Broadly, these are swallowable devices that are withdrawn from the stomach by a string, collecting cells for cytology and biomarker assays, including immunohistochemistry (IHC) and methylated DNA markers (MDM). Patients with positive results can undergo confirmatory and potentially therapeutic EGD.

**Table 2:** Novel Barrett’s esophagus screening technologies

Tool	Mode	Biomarker(s)	Reference(s)	Sensitivity	Specificity
Endosign/Cytosponge	Swallowable tethered capsule	TFF3	Ross-Ines et al [36]	79.9%	92.4%
EsophaCap	Swallowable tethered capsule	5-MDM	Iyer et al [47]	92%	94%
		5-MDM	Iyer et al [48]	93%	90%
		3-MDM	Iyer et al [49]	88%	84%
EsoCheck	Swallowable tethered balloon	2-MDM:VIM, CCNA1	Moinova et al [51]	85%	85%
			Greer et al [52]	92.9%	72.2%
			Shaheen et al [53]	87.5%	81.2%
Aeonose	Electronic nose device	Exhaled VOCs	Chan et al [54]	82%	80%
			Peters et al [55]	91%	74%
uTNE	Thin endoscope	-	Huibertse et al [58]	89%	93%
MiroCam Navi	Magnetic capsule endoscopy	-	Beg et al [62]	94%	100%
Tethered capsule endomicroscopy		-	Dong et al [63]	NA	NA
EMERALD	Serum panel	6-miRNA	Miyoshi et al [68]	82.5%	90.5%
Qin	Serum panel	5-MDM	Qin et al [69]	74%*	91%*
Liu	Serum panel	4-MDM	Liu et al [70]	66.7%*	86.3%*
Galleri	MCED	MDM	Klein et al [71]	85%**	NA
CancerGuard/CancerSEEK	MCED	ctDNA, proteins	Cohen et al [74]	~70%**	NA

*MDM,* methylated DNA marker; *NA,* not available; *TFF3,* trefoil factor family 3; *VOCs,* volatile organic compounds; * uTNE,* unsedated transnasal endoscopy; *MCED,* multi-cancer early detection; *ctDNA,* circulating tumor DNA; *miRN*A, micro RNA; *VIM,* vimentin; *CCNA1,* cyclin A1

*for esophageal adenocarcinoma

**for esophageal cancers

The EndoSign Cell Collection device (Cyted Health) consists of an encapsulated, tethered sponge, loaded on a plastic applicator. The capsule is swallowed, dissolves in the stomach, and is then retrieved by the thread, collecting esophageal cells on withdrawal for cytology and biomarker analysis. EndoSign constitutes an updated iteration of Cytosponge (Medtronic), which has been tested in combination with an assay for trefoil factor 3 (TFF3), a protein expressed on goblet cells found in IM. Cytosponge demonstrated 79.9% sensitivity for BE and 87.2% for BE segments ≥ 3 cm in the BEST2 case-control study [36]. Fitzgerald et al. published data from the BEST3 trial, in which > 13,000 primary care patients on GERD medication were randomized to Cytosponge (followed by EGD for a positive test) or usual care, with an EGD only at the discretion of their primary care provider [37]. Of 6983 patients randomized to Cytosponge, 1654 met inclusion criteria, expressed interest in Cytosponge, and successfully swallowed it. During an average follow-up of 12 months, 2% of Cytosponge patients were diagnosed with BE on EGD, including nine with dysplastic BE or stage I EAC, compared to < 1% of patients in the usual care group, with no dysplasia or stage I EAC.

Cytosponge has demonstrated good safety and tolerability. A systematic review including 2672 procedures found one episode of sponge detachment and one instance of pharyngeal bleeding [38]. Among 1488 patients in BEST3 who swallowed the Cytosponge, 60% reported gagging, although 80% were willing to repeat the procedure or recommend it to friends [39]. Cost-utility analysis of BEST3 suggested that Cytosponge is cost-effective relative to usual care for BE screening among patients with and without GERD [40, 41]. The prospect of utilizing a non-endoscopic cell collection device like EndoSign with a risk stratification tool for population screening is promising and appears cost-effective [42]. Combining Endosign with assessment for cellular atypia, aberrant p53 staining, and clinical-demographic variables may also identify BE patients at highest risk of progression to HGD/EAC, permitting the prioritization of endoscopic surveillance and reducing delays [43–46]. EndoSign is being currently researched in the United States but is not yet commercially available.

EsophaCap (Lucid Diagnostics) is another swallowable cell collection sponge paired with an MDM assay. In combination with a five component MDM assay (5-MDM), EsophaCap demonstrated high sensitivity and specificity for BE in both a multi-center case-control study and an independent test cohort [47, 48]. 3-MDM assays have yielded comparable performance, approaching 90% sensitivity and specificity for BE, with one assay 100% sensitive for HGD/EAC [49]. The test is well-tolerated, with 93% of patients willing to repeat it. EsophaCap showed comparable cost-effectiveness to Cytosponge [41]. Unfortunately, two patients suffered device detachments, one requiring endoscopic retrieval, and the US FDA issued a class 2 device recall in April 2024 [50].

EsoCheck (Lucid Diagnostics) is a swallowable balloon-based cell collection device. After the textured balloon is swallowed, it is inflated in the stomach and withdrawn, sampling esophageal cells on exit. The balloon is deflated above the gastroesophageal junction (GEJ), limiting the collection of proximal squamous mucosa. EsoCheck is paired with an MDM assay (EsoGuard) for 31 methylation sites on vimentin (VIM) and cyclin A1 (CCNA1). Recent studies have demonstrated > 85% sensitivity and 72–85% specificity for BE, as well as 100% sensitivity for EAC [51–53] Further multi-site studies are ongoing.

Swallowable cell collection devices have demonstrated promising sensitivities for BE screening and can be administered rapidly in primary care settings by non-physician staff and without sedation. They represent a tolerable and cost-effective screening option that can be followed by EGD for diagnostic confirmation and therapy. Moreover, wide-spread adoption could facilitate the expansion of screening to GERD-independent populations. Current ACG, AGA, and ESGE guidelines support non-endoscopic cell-collection devices as acceptable alternatives to sedated endoscopy for BE screening [12, 13, 16]. EndoSign (formerly Cytosponge) is used in the UK National Health Service (NHS) and received 510(k) clearance from the FDA in 2024, while EsoCheck-EsoGuard is available in the US. Lack of insurance reimbursement currently limits clinical use of both in the US.

## Exhaled Volatile Organic Compounds (VOC)

Volatile organic compounds (VOC) are gaseous metabolic byproducts. Diseases may display VOC signatures detectable in patients’ breath via an “electronic nose” device, which uses electrochemical interfaces to generate VOC profiles (“breath prints”). Studies of the Aeonose device (The eNose Company) have reported sensitivities of 82–91% and specificities of 74–80% for BE [54, 55]. Mass spectrometry-based VOC profiling for EAC detection also performed well in a cohort of 81 patients (AUROC 0.97), but its scalability is limited by cost and time investment [55, 56].

Exhaled VOC testing appears highly acceptable to patients because of its minimal invasiveness. Sijben et al. surveyed 2258 Dutch patients who were randomly assigned to 1 of 3 hypothetical EAC screening strategies and found the highest intended screening uptake for exhaled VOC testing (95%), followed by swallowable cell collection device (75%), and transnasal endoscopy (68%) (*P* < 0.001), along with the least anticipated discomfort [57]. This technology presents a promising, highly-acceptable and non-invasive option, but further clinical validation is needed prior to widespread uptake.

## Unsedated Transnasal Endoscopy

Unsedated transnasal endoscopy (uTNE) is performed with thin endoscopes (≤6.0 mm) and is highly sensitive for columnar epithelium. In a recent meta-analysis of 623 patients who underwent both uTNE and conventional EGD, uTNE showed 98% sensitivity and 99% specificity for columnar epithelium [58]. Although some uTNE endoscopes have biopsy channels, sensitivity and specificity for IM are moderately lower, which may be attributed to smaller biopsy dimensions and variable sampling protocols. uTNE is safe, with 2% of patients reporting adverse events, most commonly epistaxis and vasovagal symptoms. Although uTNE is reasonably sensitive, safe, and cost-effective, uptake has been limited, likely due to significant patient apprehension and expert-dependence [57–59].

## Esophageal Capsule Endoscopy

Pillcam ESO (Medtronic) was developed in the early 2000s, based on technology used for small intestinal capsule endoscopy [60]. Inconsistent capsule transit times, absence of insufflation to maximize visibility, and inability to obtain biopsies have been cited as limitations [61]. Beg et al. performed a pilot study using the MiroCam Navi capsule (SynMed), which incorporates magnets to guide the capsule through the esophagus and reduce variability in transit time, yielding 94% sensitivity and 100% specificity for BE [62]. This device requires further validation before it can be considered for BE screening.

## Tethered Capsule Endomicroscopy

Tethered capsule endomicroscopy (TCE) consists of a swallowable, tethered pill that uses optical coherence tomography (OCT) to generate high-resolution cross-sectional images of the esophagus. Dong et al. described their multicenter experience with TCE in 147 patients with BE, of whom 79% swallowed the capsule successfully [63]. They reported strong correlation between BE length by TCE and EGD (*r* = 0.77–0.79). Notably, patients did not have biopsy confirmation of columnar epithelia identified by TCE, and all studies were read by two experts in OCT. Although previous studies have validated the correlation between OCT findings and IM, those images were obtained during sedated endoscopy [64]. Further studies examining concordance between TCE-acquired images and biopsies are warranted before this technology can be considered for routine use.

### Serum Biomarkers

Serum biomarkers obtained from “liquid biopsies,” including microRNAs (miRNA), circulating tumor DNA (ctDNA), and protein-based assays, are appealing due to their minimal invasiveness [65]. miRNA expression profiles associate with BE and EAC [66]. Bus et al. described combinations of ≥3 miRNA which predicted BE and EAC with AUROC of 0.832 and 0.846, respectively [67]. Miyoshi et al. recently published results from the multicenter EMERALD study, which identified a 6-miRNA signature that demonstrated 82.5% sensitivity and 90.5% specificity for BE and EAC [68]. Prospective studies are needed to further validate the EMERALD assay, which is not yet commercially available.

Researchers have also investigated ctDNA methylation patterns as biomarkers for multiple cancers, including EAC. Various 4- and 5-MDM serum panels have yielded sensitivities and specificities of 67–74% and 86–91% for EAC [69, 70]. Multi-cancer early detection (MCED) tests employing ctDNA assays for a host of cancers have garnered substantial attention. A 4077-patient validation cohort in The Circulating Cell-free Genome Atlas Study using the Galleri panel (GRAIL) demonstrated 51.5% sensitivity for > 50 cancers, including 85% sensitivity for esophageal cancers [71]. Notably, sensitivity for stage I-II esophageal cancer was lower (12/25) than for stage III-IV disease (72/74). Larger prospective trials of Galleri, which costs $949 per test and is not routinely covered by insurance, are underway in the US and UK [72, 73]. CancerSEEK (now CancerGuard, ExactSciences), another MCED incorporating multiple biomarker classes, demonstrated ~ 70% sensitivity for esophageal cancers in a case control study of 1817 patients, but again with low sensitivity for stage I disease (20%) [73, 74].

While appealing due to their minimal invasiveness, the exact roles for serum biomarkers in BE/EAC screening have yet to be determined. Only MCEDs are commercially available, and guidelines do not make recommendations regarding their use. These assays likely need to demonstrate cost-effectiveness and better sensitivities for earlier-stage EAC and/or BE to be considered acceptable screening tools. Combining serum biomarkers with clinical and demographic variables has potential to enhance BE/EAC risk prediction, although studies to date have found minimal added benefit [27, 75, 76].

## Novel Barrett’s Esophagus Surveillance Tools

### Virtual/Electronic Chromoendoscopy

Because the annual risk of progression among patients with non-dysplastic BE (NDBE) is low, the detection of neoplasia is key as a threshold for EET [17]. Chromoendoscopy with either a dye or virtual/electronic system (VC) increases HGD/EAC detection compared with high-definition white light endoscopy (HDWLE) and the Seattle biopsy protocol alone. VC is more readily available and faster than dye-based systems [13]. Narrow-band imaging (NBI, Olympus) is the most widely studied VC system, although similar technologies are available on multiple platforms [11].

### Confocal and Volumetric Laser Endomicroscopy

Confocal laser endomicroscopy (CLE) uses laser and intravenous fluorescent dye to illuminate tissues, magnified to the cellular level to detect dysplastic histology [11]. Meta-analysis found that CLE yielded an additional BE-related neoplasia detection rate of 19.3% compared to NBI, although sensitivity and specificity were similar [77]. CLE is commercially available as the GastroFlex Cellvizio (Mauna Kea Technologies). ASGE guidelines recommend against the routine use of CLE for BE surveillance, citing minimal improvements in dysplasia detection and high costs [11].

Volumetric laser endomicroscopy (VLE) uses OCT within a balloon catheter to generate cross-sectional images of the esophagus with resolution sufficient to visualize dysplastic features that can be biopsied. VLE improved HGD/EAC detection rates (14% vs. 1%, *P* = 0.001) compared to Seattle protocol alone [78]. The multicenter VLE PREDICT study evaluated a computer-aided detection (CAD) algorithm which demonstrated 91% sensitivity and 82% specificity, compared to 70% sensitivity and 81% specificity exhibited by 10 VLE experts [79]. While data supporting increased dysplasia diagnosis are promising, VLE is currently limited by high costs and absent commercial availability. AGA guidelines acknowledge the potential of CLE and VLE, noting their adjunctive use in expert centers, but reinforce the need for further data prior to routine implementation [13].

### Wide Area Transepithelial Sampling with Computer-Assisted 3-Dimensional Analysis

Wide Area Transepithelial Sampling with Computer-Assisted 3-Dimensional Analysis (WATS-3D, CDx Diagnostics) intends to minimize sampling error of FB with an abrasive cytology brush that collects large sheets of tissue up to 150 μm thick, compared to 3–5 μm FB specimens [12]. A neural network reconstructs 3D images of the tissue and analyzes them for IM and dysplastic features before pathologist review. Meta-analyses have found increases in dysplasia detection of 2–7% with WATS-3D in combination with FB, although confirmation of WATS-3D dysplasia with FB was limited [80, 81]. Subsequently, Shaheen et al. published a retrospective analysis of patients with ≥2 WATS-3D samples ≥12 months apart, of whom 11.6% with WATS-3D LGD progressed to HGD/EAC with FB confirmation [82]. Patients with LGD progressed at a rate of 5.79 per 100 patient years, somewhat higher than previously reported for FB-diagnosed LGD [83]. This data suggests that LGD detected by WATS-3D as an adjunct to FB is clinically relevant, although further prospective studies are warranted to fully define its role in BE surveillance.

There is also considerable interest in WATS-3D for BE screening. A recent multicenter prospective study including 23,933 US patients with GERD symptoms who underwent WATS-3D and FB found that 19.3% of patients with ≥1 cm of columnar mucosa had IM detected only by WATS-3D [84]. Among patients with dysplasia, 44.6% were diagnosed only by WATS-3D; however, ≥50% of these patients had crypt dysplasia (CD) or indefinite for dysplasia (IND). Excluding these patients decreased the adjunctive yield for dysplasia from 80.5% to 21.6%. The authors contend that WATS-3D can be a valuable adjunct to FB for BE screening given the increased IM yield and potential to minimize repeat EGD in patients with columnar mucosa but negative FB.

WATS-3D appears to increase dysplasia detection with modest added time (4.5 min) [85]. The addition of WATS-3D to Seattle protocol was cost-effective in a BE screening model, although cost-effectiveness in surveillance has not been assessed [86]. AGA and ASGE guidelines recommend WATS-3D with Seattle protocol to maximize dysplasia detection in patients with known or suspected BE, while ESGE guidelines suggest against routine use, and ACG guidelines could not make a recommendation [11–13, 16]. Key concerns include the limited confirmation of WATS-3D dysplasia with FB and the absence of long-term clinical outcomes, raising questions of over-diagnosis and false-positives, along with the lack of comparisons to chromoendoscopy-guided FB [12, 16].

### Risk Stratification Algorithm for BE Surveillance

Current BE surveillance strategies rely on the length of BE segment and presence of dysplasia, as these predict progression to EAC [24]. As for BE screening, researchers have developed risk stratification models incorporating other variables to improve precision. Parasa et al. described the Progression in Barrett’s Esophagus (PIB) score, using sex, smoking, BE length, and LGD to assign a risk score that identified patients who progressed to HGD/EAC with a c-statistic of 0.76 [87]. Patients in low, intermediate, and high-risk groups had 0.13%, 0.73%, and 2.1% annual progression risks. External validation of the algorithm in two additional studies showed comparable performance with AUROC of 0.70–0.72 [88, 89]. AGA guidelines support the use of PIB to identify individuals under surveillance at increased risk for neoplasia and influence surveillance intervals and decisions regarding EET, although it is unclear how the score should be operationalized [13]. Prospective evaluation of the PIB score’s impact on surveillance intervals, EET thresholds, and long-term outcomes would be valuable in clarifying its utility.

### Tissue Based Biomarker Assays

Given current limitations to estimating risk of BE progression, including significant pathologist interobserver variability in diagnosing LGD, other tissue biomarkers have gained attention as potential mechanisms to improve precision [90]. p53 mutation may play an important role in neoplastic progression [91]. Meta-analysis found that p53 IHC evaluating both overexpression and loss of expression yielded odds ratios (OR) of 3.84–5.95 for progression to HGD/EAC [92]. p53 IHC may also offer diagnostic clarity, particularly among IND specimens, and AGA, ESGE, and BSG guidelines support its use for the confirmation of dysplasia, along with expert pathology review [13, 14, 16, 93]. Menon et al. reported that a p53-stratified NDBE surveillance strategy, in which patients with abnormal p53 expression had surveillance EGD at 1 year while those without had surveillance at 3 years, was cost-effective relative to conventional surveillance [94]. p53 antibodies are available from several vendors, although clinical use remains limited due to interobserver variability in interpretation [95].

TissueCypher (Castle Biosciences) analyzes signals from multiple biomarkers, including p53, on biopsies to generate risk scores for progression to HGD/EAC [96]. Pooled analysis of 552 patients with NDBE, IND, or LGD found that a high score increased risk of progression (OR 6.0), particularly among those with NDBE (OR 14.23) [97]. TissueCypher demonstrated 71.4% sensitivity for progression to HGD/EAC among 154 patients with community-diagnosed LGD, compared to 63.2% sensitivity of 30 expert and community pathologists [98]. TissueCypher detected a mean of 43% of progressors considered low-risk (i.e. downgraded to NDBE) by pathologists and increased sensitivity for progressors to 80.4%. The authors proposed that patients with community-diagnosed LGD and intermediate- or high-risk scores could be offered EET or < 1 year surveillance, while patients with confirmed LGD or IND and low-risk scores could be offered < 1 year surveillance. TissueCypher was also effective in predicting prevalent neoplasia (i.e., HGD/EAC on repeat EGD < 1 year), for which high-risk patients had OR 46.0 [99]. In a modeling study, TissueCypher was cost-effective after five years at a sensitivity of ≥50.6% [100]. A decision-making analysis found that management was escalated for 21.7% of patients with NDBE, IND, or LGD, with a change from surveillance-only to EET or a shorter surveillance interval [101]. Likewise, 33.4% of patients had management de-escalated from EET to surveillance-only or an extended surveillance interval. There are no prospective clinical trials showing differences in outcomes in using TissueCypher for clinical decision making. AGA guidelines support the use of TissueCypher for risk stratification among patients with NDBE, while ACG guidelines could not make a recommendation regarding the use of tissue biomarkers, citing limited performance characteristics [12, 13].

BarreGEN (Interpace Diagnostics) quantifies biopsies’ mutational load at tumor suppressor genes that associate with BE-related neoplasia [102]. In a 69-patient case-control study, BarreGEN demonstrated AUROC of 0.95 for progression to HGD/EAC, but a subsequent validation study found poor discrimination ( AUROC 0.50), which the authors attributed to the use of crude lysates rather than purified DNA [103]. Trindade et al. evaluated BarreGEN among 28 patients with IND, of whom two developed HGD, reporting 100% sensitivity and 85% specificity for progression [104]. This assay needs further validation in larger studies to clarify its utility in BE risk stratification.

Finally, Laun et al. recently published the results of a 240-patient multicenter study validating Esopredict (Previse), an MDM assay performed on formalin-fixed biopsies combined with age to predict five-year progression to HGD/EAC [105]. Prevalence-adjusted HGD/EAC risk was 21.5% among patients with high-risk scores, compared to 1.85% among low-risk patients. The authors proposed shortening surveillance intervals for patients with NDBE and high-risk scores, which constituted 14 of 26 (57.7%) NDBE patients who progressed. Further validation studies of Esopredict are ongoing to help define its optimal role in BE risk stratification.

## Conclusion

The incidence of EAC continues to rise, despite screening programs aimed at the detection of its precursor, BE, and surveillance with effective EET for early neoplasia. Most patients with EAC are diagnosed outside of screening and surveillance programs and have poorer outcomes. Screening strategies that rely on the presence of GERD are insufficiently sensitive, while GERD-independent approaches compromise specificity. EGD is the diagnostic standard, but its costs and invasiveness limit screening uptake. BE/EAC risk calculators incorporating variables available in the EHR may enhance risk assessment and inform the decision to pursue screening. Non-endoscopic screening tools, including swallowable cell collection devices, have shown promise as cost-effective alternatives to conventional EGD and may increase screening uptake. Serum biomarker assays, including MCEDs, are compelling due to their minimal invasiveness, but will require improved performance characteristics and further validation. Collaboration with primary care providers to implement these novel tools for identifying and screening at-risk patients is critical.

Given the low overall risk of progression to HGD/EAC, the identification of patients at highest risk is key to minimizing both over- and under-surveillance. WATS-3D may increase dysplasia yield, although the lack of comparisons to VC-guided FB and high proportion of WATS-3D-only LGD, IND, and CD have been cited as barriers to widespread uptake. Algorithms incorporating clinical and histopathologic variables, such as the PIB score, may enhance risk assessment and the choice of surveillance interval. Finally, tissue-based biomarker assays, including p53 IHC, TissueCypher, and Esopredict, may aid in identifying high-risk patients, particularly among those with NDBE or community-diagnosed LGD. Further prospective data will clarify these technologies’ roles in BE management [106].

## Key References

- Rubenstein JH, Fontaine S, MacDonald PW, Burns JA, Evans RR, Arasim ME, et al. Predicting Incident Adenocarcinoma of the Esophagus or Gastric Cardia Using Machine Learning of Electronic Health Records. Gastroenterology. 2023;165(6):1420-9.e10. 10.1053/j.gastro.2023.08.011.
  - ○  The K-ECAN risk prediction tool was developed and internally validated using a large data set from the Veterans Health Administration Corporate Data Warehouse. The tool, which incorporates electronic health record-based demographic, medication, laboratory, and comorbidity data, demonstrated AUROC of 0.77 for prediction of incident esophageal adenocarcinoma and gastric cardia adenocarcinoma, exceeding that of other risk prediction tools published to date. This tool has the potential to significantly enrich the BE screening population once it can be validated outside of Veteran populations and integrated with the electronic health record(s).
- Shaheen NJ, Othman MO, Taunk J, Chang KJ, Jaganmohan S, Yachimski PS, et al. Use of a Two-gene Methylated DNA Biomarker Assay and Non-Endoscopic Balloon for Detection of Barrett's Esophagus among High-Risk Individuals in a Screening Population. Am J Gastroenterol. 2024. 10.14309/ajg.0000000000003238.
  - ○  EsoCheck-EsoGuard (Lucid Diagnostics) was tested prospectively in a multicenter cohort of 180 men, > 50 years old with chronic GERD, 93 of whom underwent upper endoscopy and contributed to the primary analysis. EsoCheck-EsoGuard was 87.5% sensitive and 81.2% specific for BE, with negative predictive value of 98.6%. This study highlights the potential of EsoCheck-EsoGuard as a non-endoscopic screening alternative in at-risk patients.
- Miyoshi J, Mannucci A, Scarpa M, Gao F, Toden S, Whitsett T, et al. Liquid biopsy to identify Barrett's oesophagus, dysplasia and oesophageal adenocarcinoma: the EMERALD multicentre study. Gut. 2024. 10.1136/gutjnl-2024-333364.
  - ○  The EMERALD multicenter case-control study identified a 6-miRNA serum signature that demonstrated AUROC 91.9% with corresponding 82.5% sensitivity and 90.5% specificity for BE and EAC among a 295-patient validation cohort. The assay performed particularly well among patients with GERD (AUROC 94.8%, with sensitivity and specificity of 92.8% and 85.1%, respectively. This serum-based assay holds promise as a minimally invasive screening test but will require further prospective validation.
- Laun SE, Kann L, Braun J, Gilbert S, Lunz D, Pierre F, et al. Validation of an Epigenetic Prognostic Assay to Accurately Risk-Stratify Patients with Barrett's Esophagus. Am J Gastroenterol. 2024. 10.14309/ajg.0000000000003030.
  -  ○  Esopredict (Previse), a tissue-based biomarker assay assessing methylation signals from biopsy specimens, was evaluated among 240 patients with BE. Patients were divided in to one of four risk stratifaction groups based on assay activity. Prevalence-adjusted HGD/EAC risk was 21.5% among patients with high-risk scores, compared to 1.85% among low-risk patients. The study highlights the potential for adjustment of surveillance interval based on findings beyond dysplasia and segment length, as the authors proposed shortening surveillance intervals for patients with NDBE and high-risk scores, which constituted 57.7% of NDBE patients who progressed.
- Iyer PG, Codipilly DC, Chandar AK, Agarwal S, Wang KK, Leggett CL, et al. Prediction of Progression in Barrett's Esophagus Using a Tissue Systems Pathology Test: A Pooled Analysis of International Multicenter Studies. Clin Gastroenterol Hepatol. 2022;20(12):2772-9.e8. 10.1016/j.cgh.2022.02.033.
  - ○  TissueCypher (Castle BioSciences), a tissue-based assay which analyzes signals from multiple biomarkers, including p53, to generate a risk score, was evaluated in this pooled analysis of 552 patients with NDBE, IND, or LGD. The authors found that a high score increased risk of progression (OR 6.0), particularly among those with NDBE (OR 14.23). Sensitivity and specificity were 38% and 94%, respectively. These results suggest that TissueCypher may add to risk assessment among patients undergoing BE surveillance, when added to pathologist review and clinical variables, although limited sensitivity remains a concern.

## Data Availability

No datasets were generated or analysed during the current study.
